# The composition and predictive function of the fecal microbiota in female donkeys across different reproductive cycles

**DOI:** 10.3389/fmicb.2025.1565360

**Published:** 2025-06-25

**Authors:** Jingya Xing, Mingquan Jia, Guoliang Zhang, Lanjie Li, Shuai Liu, Guangyu Li, Guiqin Liu

**Affiliations:** ^1^College of Animal Science and Technology, Qingdao Agricultural University, Qingdao, China; ^2^College of Agronomy, Liaocheng University, Liaocheng, China; ^3^Laboratory of Gastrointestinal Microbiology, National Center for International Research on Animal Gut Nutrition, Nanjing Agricultural University, Nanjing, China

**Keywords:** female donkey, different reproductive cycles, fecal microbiota, microbial communities, functional prediction

## Abstract

The microorganisms residing in the gastrointestinal tract of monogastric herbivores play a vital role in nutrient absorption and maintaining the host’s health. However, the quantitative and functional establishment of these microorganisms in female donkeys across different reproductive cycles has not yet been examined. Knowledge regarding the composition and function of gut microbiota in female donkeys during different reproductive cycles remains limited. By applying high-throughput sequencing technology and functional prediction applied to fecal samples from female donkeys across different reproductive cycles, we characterized their gut microbial composition and predicted their functional profiles. The fecal microbiota diversity in female donkeys showed no significant differences across different reproductive cycles through alpha diversity. However, the relative abundance of *Firmicutes* was higher during lactation, whereas *Bacteroidetes* were significantly higher during pregnancy. Principal coordinate analysis (PCoA) revealed the gut microbiota composition of pregnant female donkeys differed significantly from that in lactating and non-pregnant female donkeys. *Bacteroidetes* and *Alloprevotella* dominated during pregnancy in donkeys, while *Firmicutes* and unidentified *Clostridiales* were more prevalent during lactation. For functional prediction, there were significant differences in the relative abundance of pathways in the feces of female donkeys across different reproductive cycles, such as immune system processes, metabolism, glycan biosynthesis and metabolism, environmental adaptation and cell motility (*p* < 0.05 or *p* < 0.01). By correlating metabolic functions with microbial phyla, we suggest that metabolic and immune functions associated with the gut microbiota in lactating donkeys may be reduced compared to pregnant donkeys. Principal component analysis (PCA) revealed that the functional KEGG Orthologs (KOs) in the fecal microbiota of pregnant donkeys were distinctly separated from the lactation and non-pregnant female donkeys. Microbial community composition and structure exhibit distinct characteristics across different reproductive cycle, which are closely related to the functions of the microbiome. Our findings provide a foundation for understanding the compositional and functional differences in the microbial communities of mares’ feces across different reproductive cycles, offering valuable insights for the precise feeding of mares throughout different reproductive cycles.

## 1 Introduction

Owing to its remarkable ability to influence the host’s physiology, the gut microbiome has been termed a “forgotten organ” (Cai et al., 2023). The gut microbiota in animals plays a critical role in physiological processes such as energy metabolism (Amabebe et al., 2020; Chen et al., 2020), immune function (Cai et al., 2024; Zhang et al., 2024), behavior (Awe et al., 2024), cell proliferation (Dougherty et al., 2020), and differentiation (He et al., 2024). Typically, microorganisms form a protective barrier by colonizing the gastrointestinal tract, maintaining the dynamic balance of digestive tract microorganisms to support digestion, nutrient absorption, and overall health. However, its composition is influenced by diet (Ross et al., 2024), environment (Seo, 2024), exercise behavior and host genetics (Clark and Mach, 2016), and fetal development (Rao et al., 2021). Maintaining a balanced gastrointestinal microbiota is essential for animals to preserve good health and sustain optimal production performance. Microorganisms help the body resist pathogenic invasions, promote immune system development (Donald and Finlay, 2023), assist in nutrient digestion and absorption (Bahaddad et al., 2023), and provide energy to the host through metabolic byproducts (Steinbach et al., 2024). Their composition and changes directly affect the body’s health and physiological functions. If the gut microbiota becomes imbalanced, it may lead to gastrointestinal or even systemic diseases.

Animals undergo significant hormonal, metabolic, immune, and nutritional adjustments during different reproductive cycles to ensure the healthy development of their offspring. These changes can disrupt the balance of microbial communities, potentially leading to disease (Avellar et al., 2019). During normal pregnancy, excessive obesity and reduced insulin sensitivity support fetal growth and prepare the body for lactation (Dicks et al., 2014). However, pregnancy-related obesity is linked to shifts in gut microbiota composition (Gohir et al., 2015). Contradictory studies suggest that the gut microbiota in humans remains stable during pregnancy (Jost et al., 2014), likely due to uncontrollable factors such as dietary habits or antibiotic use during research. Maternal microorganisms can transfer to the fetus via the placental barrier, playing a critical role in the offspring’s immunity during lactation (Liu et al., 2024; Macpherson et al., 2017). Studies show that offspring from germ-free mothers have compromised immune system development, emphasizing the importance of maternal microorganisms for offspring health during pregnancy (Muglia et al., 2022).

The various stages of the maternal reproductive cycle are interrelated, and the microbial community in one stage can affect the reproductive ability in the subsequent stage or even for life. Therefore, understanding the changes in gut microbiota during the reproductive cycle of healthy mothers is particularly important for breeding livestock and poultry. Gut microbiota play a vital role in maintaining host immunity, metabolic balance, and nutrient absorption. Comparative analysis across different reproductive stages can help identify key microbial communities potentially associated with immune function, energy metabolism, and anti-inflammatory responses, thus providing a theoretical foundation for safeguarding the health of female donkeys. In this research, we employed high-throughput sequencing technology combined with the PICRUSt method to characterize the succession pattern and metabolic function changes of gut microbiota throughout the reproductive cycle of female donkeys. While most studies on gut microbial diversity across reproductive cycles have concentrated on common livestock species, systematic investigations in female donkeys remain limited. Considering the substantial differences in nutritional demands during various reproductive stages, exploring the gut microbiota of female donkeys can provide valuable insights to inform nutritional strategies and precision management.

## 2 Materials and methods

### 2.1 Animals, management and sample

Fifteen healthy Dezhou female donkeys, approximately 6 years old (± 3 months) with an average body weight of 250 ± 10 kg, were selected for the experiment. They were divided into three groups (pregnant, non-pregnant, and lactating) based on their different reproductive cycles. The donkeys were housed at the National Black Donkey Breeding Center, raised by Dong-E–E-Jiao Co., Ltd. (Dong’e County, Shandong Province). Throughout the feeding period, the donkey house was kept clean, and all equipment was regularly disinfected to maintain a hygienic environment. All donkeys were free from digestive tract diseases for at least 3 months prior to sampling. They were provided a standard concentrate diet equivalent to 1.3% of their body weight, administered twice daily (at 8 am and 4 pm). All donkeys were fed the same diet, including supplements, vitamins, and other components. Additionally, they had ad libitum access to fresh water and a forage mix of soybean straw in a 60:40 ratio.

The composition and nutritional content of the concentrate feed are presented in Table 1. Beanstalk was used as the roughage, with its nutritional profile shown in Table 2. The experiment was initiated during the mid-gestation period of the donkeys (months 5 to 8 of pregnancy), with samples collected from early pregnancy through the 6th month.

**Table 1:** The composition and nutritional content of the concentrate feed /%.

Items	Contents
Ingredients	
Corn	40.00
Soybean meal	27.00
Wheat bran	21.00
Stone power	5.00
Calcium hydrogen phosphate	1.60
Salt	0.70
Premix^a^	4.7
Toal	100.00
Nutrient level^b^	
CP	18.20
CF	3.73
EE	2.79
Ca	2.36
P	0.74
DE/(Mcal/kg)	2.78

a Premix provides per kg of feed: VE 50 mg, VA 20,000 IU, VK 2.5 mg, VD 3,500 mg, VB1 2.5 mg, VB2 8.0 mg, VB3 25 mg, VB5 32 mg, VB6 0.5 mg, VB12 50 μg, Folic Acid 0.5 mg, biotin 90 ug, Fe 200 mg, Mn 50 mg, Zn 220 mg, Cu 30 mg, Se 0.45 mg, I 2.0 mg.

b CP, crude protein; EE, crude extract; CF, crude fiber; NDF, neutral detergent fiber; ADF, acid detergent fiber; DE, digestibility energy. DE was a calculated value, while the others were measured values.

**Table 2:** The nutrient level of beanstalk /%.

Nutrient levels	Contents
CP	5.31
EE	0.39
CF	43.39
ADF	53.53
NDF	64.20
DE/(MJ/kg)	2.65

NDF, neutraldetergent fiber; ADF, acid detergent fiber; DE, digestibility energy.

These experimental female donkeys were fasted for 3 h before sampling. After feeding for 1 h, rectal content samples were collected by farm technicians wearing sterile gloves. The samples were collected in 50 mL sterile cryopreservation tubes and immediately preserved in liquid nitrogen. Subsequently, they were transported to the laboratory and stored at −80°C. The samples of pregnant female donkeys were labeled as P, non-pregnant female donkeys as NP, and lactating female donkeys as L, with each physiological state representing a separate sample group.

### 2.2 Sample processing and high-throughput sequencing

Transfer the frozen rectal content samples from −80°C to 4°C for 1 h to defrost. Take 10 g of the sample and mix it evenly on a clean experimental bench for further use. Total DNA was extracted and purified from the processed samples using the QIAamp Stool Mini Kit (Qiagen, Valencia, CA, USA) following the manufacturer’s protocol. The DNA’s purity and integrity were evaluated using a Nanodrop 2000 Spectrophotometer (Nanodrop, Wilmington, DE, USA). The samples were stored at −20°C for future use. PCR amplification was performed on the V3-V4 regions of the bacterial 16S rRNA gene using primers 341F (5’-ACTCCTACGGGAGGCAGCAG-3’) and 806R (5’-GGACTACHVGGGTWTCTAAT-3’). Library construction, Qubit quantification, and library testing were carried out as previously described (Xing et al., 2020). Finally, single-end sequencing was performed using the Ion S5™ XL system (Thermo Fisher Scientific) with a read length of SE600, generating a total of 1,245,890 reads.

### 2.3 Bioinformatics analysis

To improve the accuracy and reliability of the analysis results, the raw data were first spliced and filtered to obtain clean data. Low-quality reads were filtered using Cutadapt (v1.9.1), and barcode and primer sequences were trimmed with Chromas. Sequence assembly was performed using DNASTAR, and chimeric sequences were identified and removed using Bellerophon. All clean reads from the samples were clustered into OTUs (Operational Taxonomic Units) using Uparse (v 7.0.1001) (Haas et al., 2011), with a default identity threshold of 97% for sequence similarity. OTUs were taxonomically annotated using the Mothur method in conjunction with the SILVA132 database (Edgar, 2013), with a confidence threshold set between 0.8 and 1. Taxonomic information was obtained, and the community composition of each sample was analyzed at different taxonomic levels. Finally, the data from all samples were normalized based on the sample with the lowest sequencing depth. Subsequent alpha diversity and beta diversity analyses were conducted using the normalized data. The results were visualized using Venn and petal diagrams, providing insights into species richness and evenness within samples, as well as identifying shared and unique OTUs among different groups. Alternatively, OTUs were aligned using multiple sequence alignment to construct a phylogenetic tree. We detected statistically significant differences in alpha diversity, and evaluated microbial composition in female donkeys across different reproductive cycles using one-way ANOVA. Unifrac distances were calculated and UPGMA sample clustering trees were constructed using QIIME (v 1.9.1) (Langille et al., 2013). PCA, PCoA, and NMDS plots were generated using R software (v 2.15.3). LEfSe analysis was performed using the LEfSe software, with the default linear discriminant analysis (LDA) values threshold set at 4. Metastats analysis was conducted using R software to perform permutation tests between groups at each taxonomic level, generating *p*-values, which were then adjusted to obtain q-values. The specific data processing and software tools used were the same as those described previously (Xing et al., 2020).

### 2.4 PICRUSt analysis

To uncover the functional potential of the gut microbiota, PICRUSt (v1.0.0) was utilized to predict the metabolic capabilities of microbial communities using the OTUs table as a reference in QIIME (v 1.9.1) software (Langille et al., 2013). Subsequently, PICRUSt was utilized to predict functional pathways based on Kyoto Encyclopedia of Genes and Genomes (KEGG) annotations at levels 1 and 2. The abundances of functional categories were calculated using OTU abundance data, and correlations between differential microbial taxa and specific metabolic pathways were analyzed. The relationships between functional capacities and predicted relative gene abundances were evaluated using PCoA and heatmaps. Changes in relative abundances between different groups were compared using LSD-t tests.

## 3 Results

### 3.1 OTUs analysis and alpha diversity

**Figure 1:**
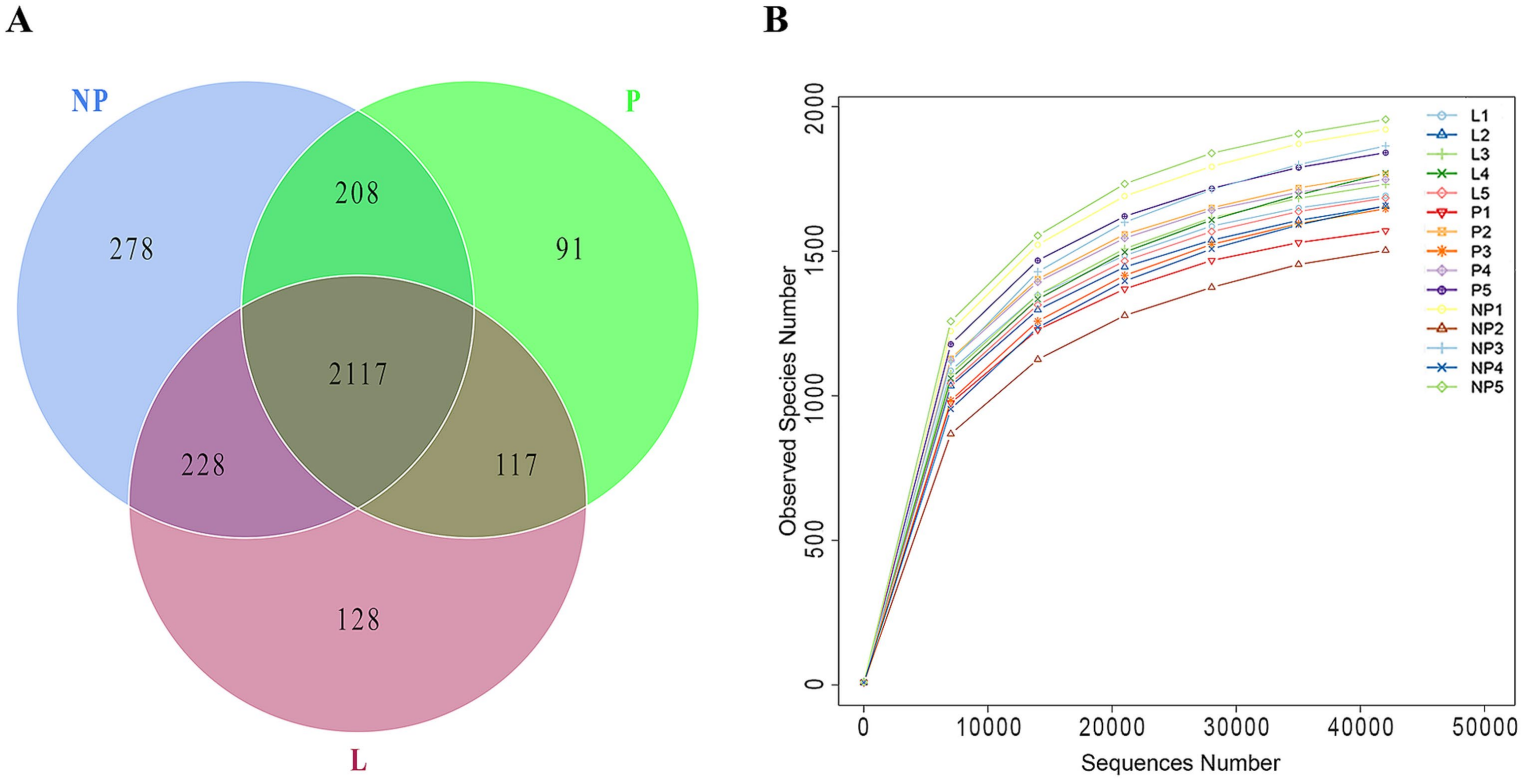
Bacterial OTUs detected in fecal microbiota of female across different reproductive cycles. **(A)** The Venn diagram highlights the shared OTUs among different groups, with overlapping areas indicating common OTUs. **(B)** The rarefaction curve illustrates the number of OTUs detected across varying sequencing depths. P, pregnant female donkeys; NP, non-pregnant female donkeys; L, lactating female donkeys.

All samples underwent chimeric filtration of the sequencing results, yielding a total of 1,655,064 valid sequences. The sequences were grouped into 2,117 OTUs based on 97% sequence similarity. A total of 2,117 core OTUs were identified across all samples, with 497 OTUs unique to each reproductive period. There were 91 OTUs unique to group P, 278 OTUs unique to group NP, and 128 OTUs unique to group L (Table 3 and Figure 1A). The rarefaction curves for all samples leveled off at a sequencing depth of 42,028, suggesting that this depth adequately captured the majority of microorganisms. As shown in Table 3, there were no significant differences in gut microbiota diversity among female donkeys across the various reproductive cycles (*p* > 0.05). Notably, the number OTUs and species richness in group L were the lowest (Figure 1B).

**Table 3:** Microbial annotation results and OTUs analysis in fecal microbiota of female donkeys across different reproductive cycles.

Group	OTUs	Phylum	Class	Order	Family	Genus
P	2,681	24	33	61	97	160
NP	2,658	24	32	59	91	142
L	2,938	24	32	57	94	171

P, pregnant female donkeys; NP, non-pregnant female donkeys; L, lactating female donkeys.

Sequencing of 15 rectal content samples revealed no significant differences in the effective sequences obtained from rectal content samples across different reproductive cycles (*p* > 0.05). At a sequencing depth of 42,028, the Good’s coverage indices were higher than 0.99, indicating that over 99% of all bacterial taxa were represented in all samples. The species richness indices, including observed species, Chao1, and Ace, showed no significant differences (*p* > 0.05). The Shannon diversity indices exceeded 8.5, and the Simpson indices were all greater than 0.99. Furthermore, no significant differences were observed among the three groups (*p* > 0.05), suggesting that the female donkeys harbored a complex gut microbiota at each stage of their reproductive cycle (Table 4).

**Table 4:** Analysis of α diversity indexes in fecal microbiota of female donkeys across different reproductive cycles.

Items	Groups			*p*-value
	P	NP	L	
Good^’^s coverage (mean ±SE)	0.99 ± 0.00	0.99 ± 0.00	0.99 ± 0.00	0.293
Chao 1 (mean ±SE)	1837.02 ± 78.42	1957.94 ± 83.98	1889.50 ± 78.42	0.523
Ace index (mean ±SE)	1855.65 ± 47.09	1968.43 ± 77.63	1885.83 ± 52.46	0.421
Observed species (mean ±SE)	1714.80 ± 47.45	1780.40 ± 86.62	1706.60 ± 19.87	0.620
Shannon (mean ±SE)	8.88 ± 0.13	8.59 ± 0.20	8.70 ± 0.11	0.394
Simpson (mean ±SE)	0.99 ± 0.00	0.99 ± 0.03	0.99 ± 0.00	0.174

Identical superscripts or no superscript, whether lowercase or uppercase, indicate no significant difference (*p* > 0.05).

### 3.2 Taxonomic composition of fecal microbiota among female donkeys across different reproductive cycles

**Figure 2:**
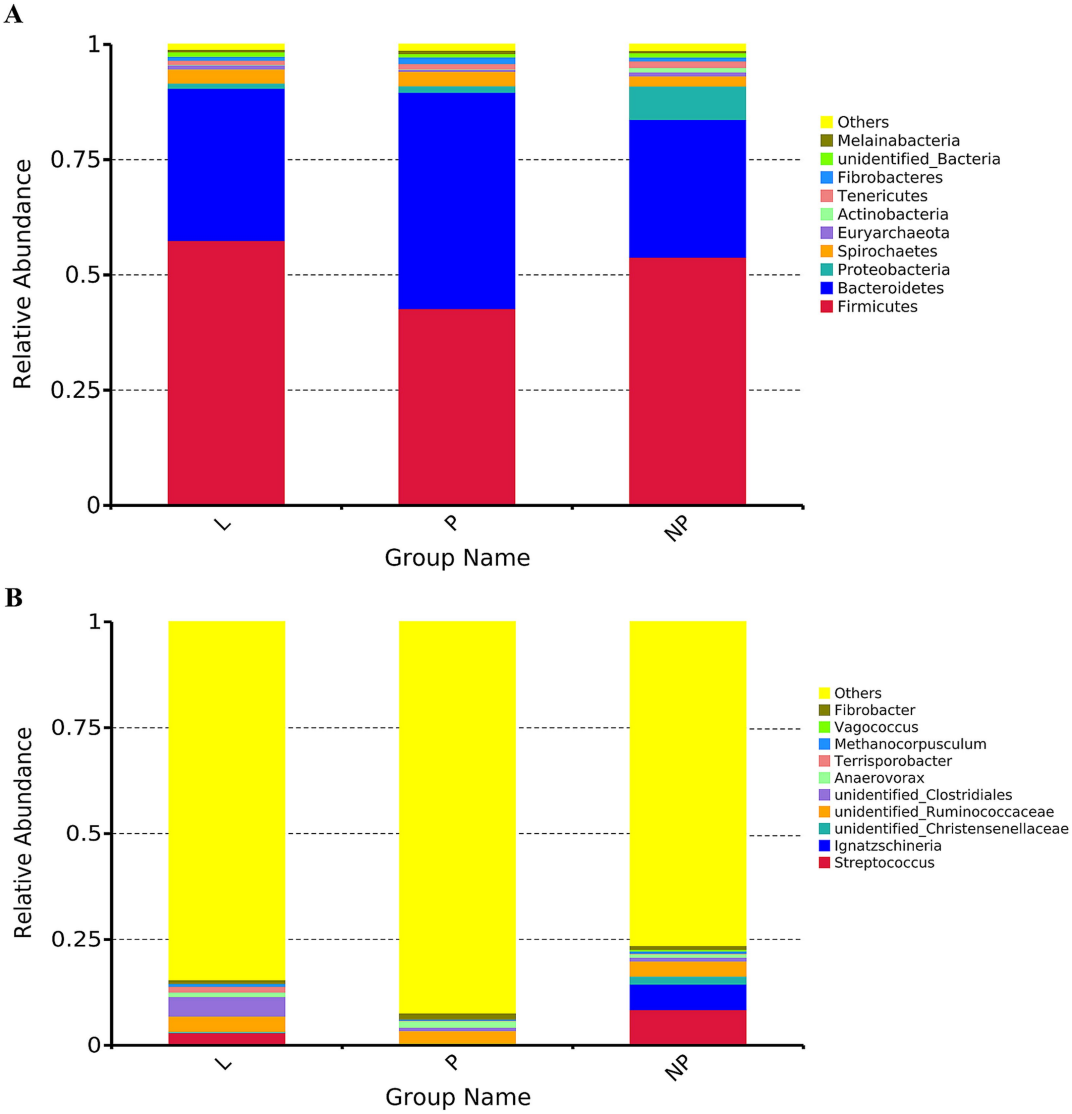
Microbial community structure in fecal microbiota of female donkeys across different reproductive cycles, displayed by taxonomic composition at the phylum level **(A)** and genus level **(B)**.

**Figure 3:**
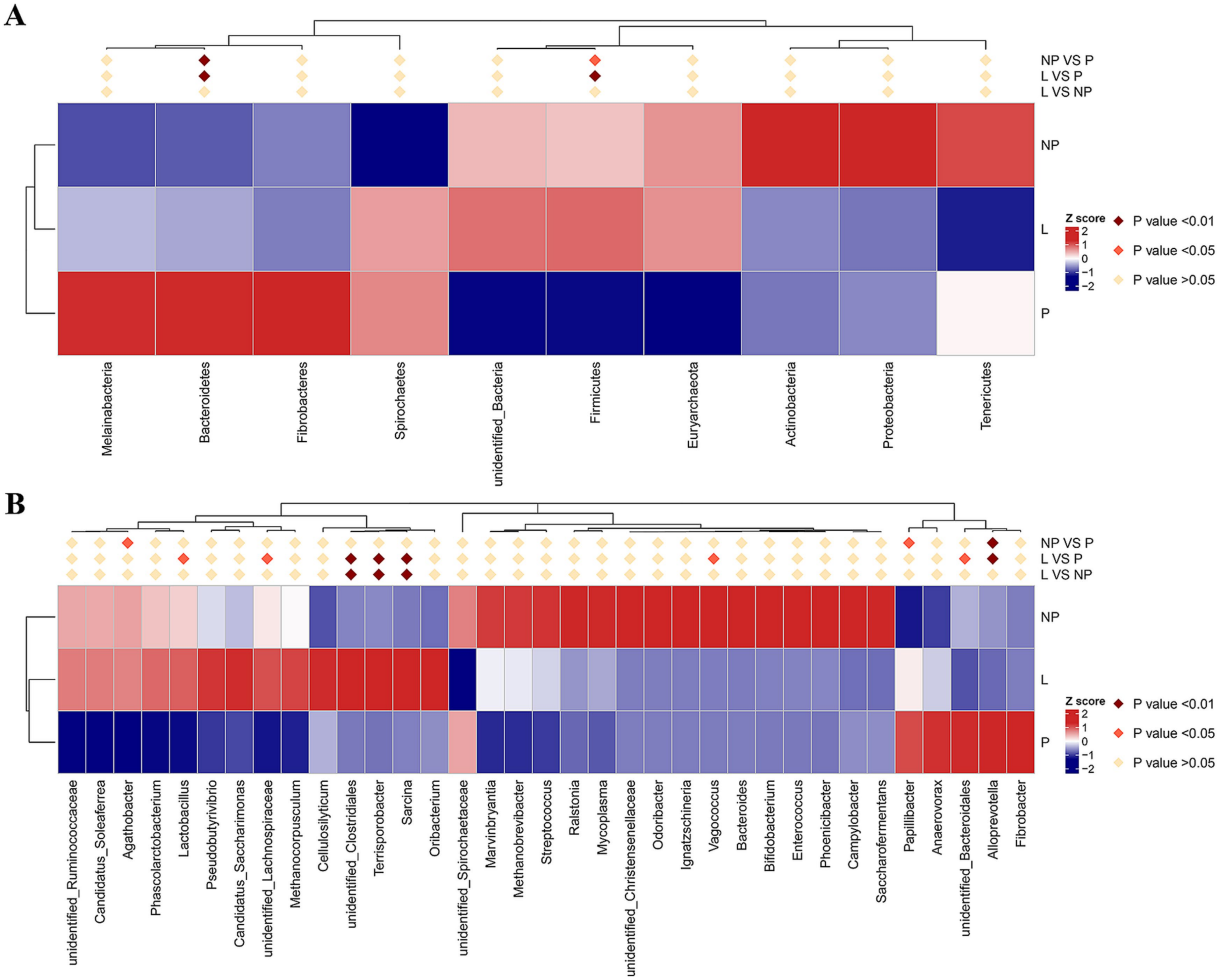
MetaStat heat maps in fecal microbiota of female donkeys across different reproductive cycles, presented at the phylum level **(A)** and genus level **(B)**. Differences in relative abundance of each functional gene were examined using *T*-test, and the *p*-value <0.05 suggests a significant difference; the *p*-value <0.01 indicates a highly significant difference; the *p*-value > 0.05 indicates that the difference is not significant.

After species annotation of each group based on OTUs, the relative abundance of microorganisms at the phylum and genus levels was analyzed (Figure 2). To further investigate the differential species of gut microbiota in female donkeys across different reproductive cycles, we selected the top 10 or top 35 phyla or genera based on their abundance ranking. Using species annotations and abundance data at the phylum and genus levels, Metastats was employed to cluster the samples and generate a heatmap based on species and sample levels (Figure 3).

The top 10 bacterial phyla in the fecal samples were identified and analyzed (Figure 2A). *Firmicutes* and *Bacteroidetes* were the predominant phyla across all groups. In group L, *Firmicutes* and *Bacteroidetes* accounted for 57.5 and 33.0%, respectively; in group P, they accounted for 42.7 and 47.0%; and in group NP, they accounted for 53.8 and 29.9%, respectively. Differential analysis using Metastats showed that the phylum *Bacteroidetes* in the pregnancy group was significantly higher than in the other groups (*p* < 0.01). The phylum *Firmicutes* was significantly higher in the lactation group compared to the pregnancy group (*p* < 0.01), and the non-pregnancy group was significantly higher than the pregnancy group (*p* < 0.05) (Figure 3A). Notably, there were high variability in gut microbiota composition among individuals in the non-pregnancy group, with the relative abundance of *Proteobacteria* ranging from 2 to 31%.

At the genus level, genera with relative abundances less than 1% were clustered into an “Others” category. The results revealed significant differences among the three treatment groups (*p* < 0.05) (Figure 2B). The genus with the highest relative abundance was identified as *Streptococcus*, accounting for 0.09, 8.4, and 3% in the P, NP, and L groups, respectively. Its presence was primarily concentrated in the intestines of non-pregnant female donkeys. Other genera with notable abundance differences included *Ignatzschineria*, which accounted for 0.03% in both the P and L groups, and 6.1% in the NP group; Unidentified *Christensenellaceae*, representing 0.25% in the P and L groups, and 1.9% in the NP group; and Unidentified *Ruminococcaceae*, which made up 3.2, 3.6, and 3.7% in the P, NP, and L groups, respectively. Similarly, unidentified *Clostridiales* contributed 0.7% in the P group, 0.9% in the NP group, and 4.6% in the L group. *Anaerovorax* accounted for 1.6, 0.8, and 1.1% in the P, NP, and L groups, respectively. *Terrisporobacter* showed proportions of 0.06% in the P group, 0.1% in the NP group, and 1.37% in the L group, while *Methanocorpusculum* represented 0.7, 0.3, and 0.5% in the L, P, and NP groups, respectively. *Vagococcus* was present at 0.003 and 0.438% in the L and NP groups, respectively, but was undetected in the P group. Finally, *Fibrobacter* appeared at 0.84% in the NP and L groups, and 1.43% in the P group (Figure 2B).

As illustrated in Figure 3B, the relative abundance of *Alloprevotella* in the P group was significantly higher than in the other two groups (*p* < 0.01). The unclassified *Bacteroidales*, *Vagococcus*, and *Papillibacter* were significantly higher in the L and NP groups, respectively (*p* < 0.05). Additionally, the unclassified genus *Sarcina*, *Terrisporobacter*, and unclassified *Clostridiales* were significantly higher in the L group compared to the P and NP groups (*p* < 0.01), and unclassified *Lachnospiraceae* and *Lactobacillus* in the L group were significantly higher than in the P group (*p* < 0.05). Furthermore, the relative abundance of *Agathobacter* in the NP group was significantly higher than in the P group (*p* < 0.05).

**Figure 4:**
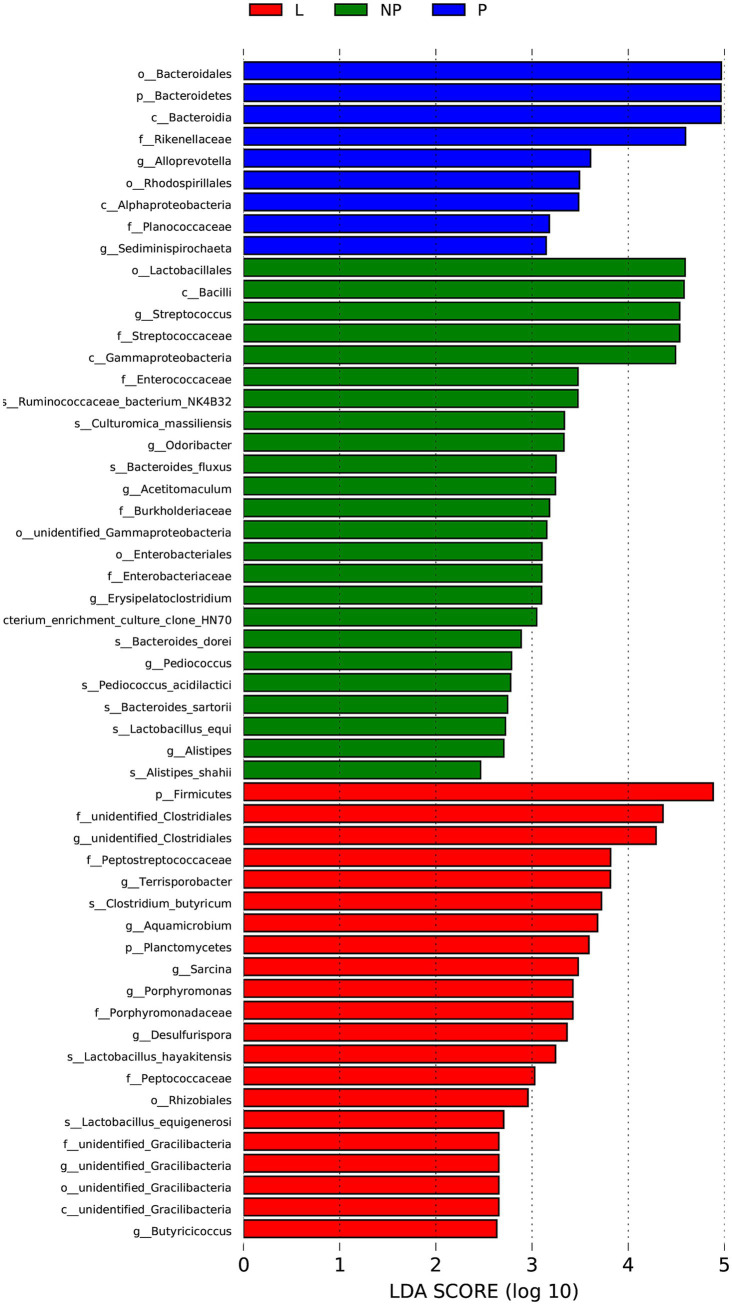
Histogram of species distribution with significant differences in fecal microbiota of female donkeys across different reproductive cycles (LDA > 2). The horizontal axis represents the effect size of each species (LDA value), while the vertical axis denotes the different species.

To identify bacteria with significant differences in abundance across different reproductive stages, we combined the results of LEfSe and Metastats for further analysis. Based on the analysis criteria (LEfSe, *p* > 0.05 and LDA > 2; Metastats, *p* < 0.05 and Q < 0.1), 2 phyla and 14 bacterial species were selected. The abundance of p_*Firmicutes* and f_unidentified *Clostridiales* in the L group was significantly higher than in the P and NP groups, while f_*Peptostreptococcaceae* was significantly higher in the pregnant female donkeys (*p* < 0.05). The abundance of g_*Alloprevotella* and c_*Alphaproteobacteria* in the P group was significantly higher than in the NP and L groups (*p* < 0.05), while p_*Bacteroidetes*, c_*Bacteroidia*, and o_*Bacteroidales* were significantly higher in the L group (*p* < 0.05). *Bacteroidetes*, g_*Alloprevotella* and c_*Alphaproteobacteria* might be dominant during pregnancy in donkeys, while p_*Firmicutes* and f_unidentified *Clostridiales* prevailed during lactation. This suggests that *Bacteroidetes*, g_Alloprevotella and c_*Alphaproteobacteria* may be associated with pregnancy, while p_*Firmicutes* and f_unidentified *Clostridiales* may be linked to lactation (Figure 4).

### 3.3 Comparative analysis of gut microbiota between female donkeys’ feces across different reproductive cycles (beta diversity)

**Figure 5:**
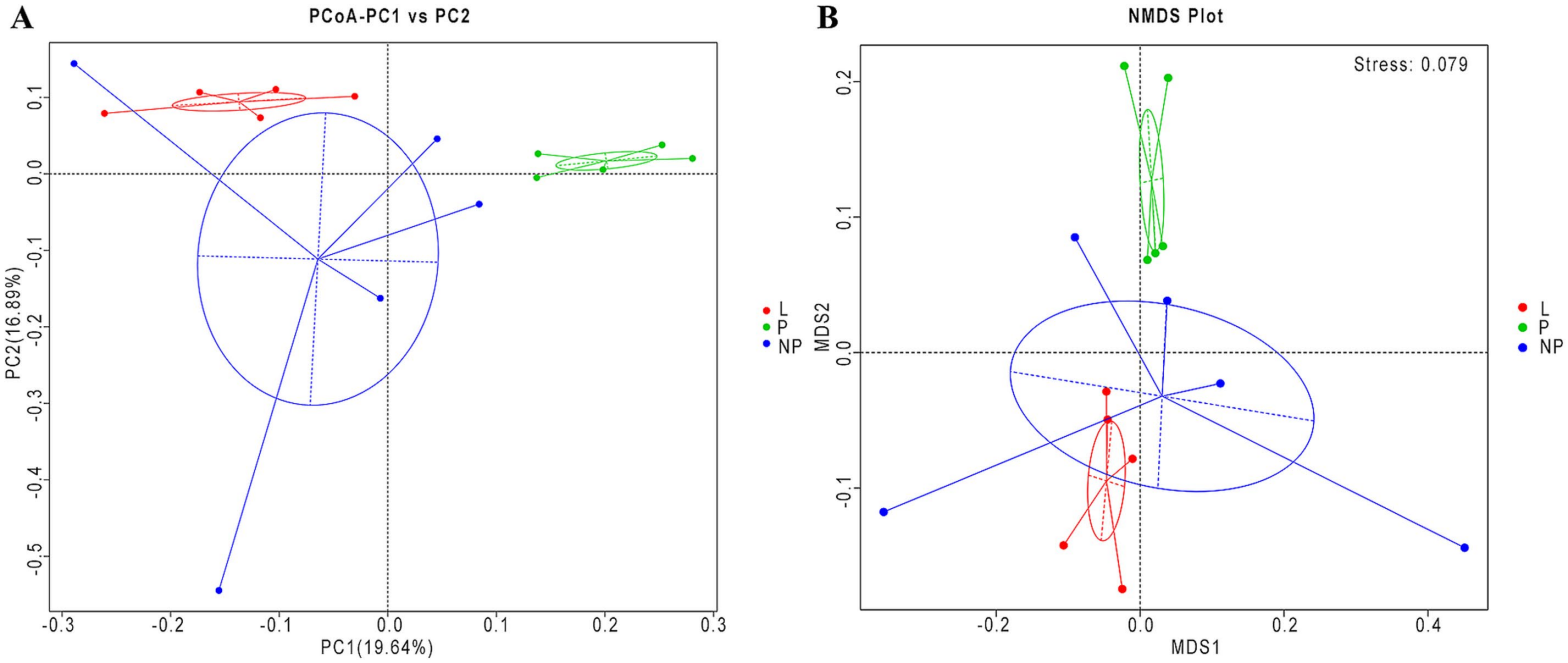
Beta diversity of the fecal microbiota in female donkeys across different reproductive cycles. Principal component analysis of female donkeys’ feces across different reproductive cycles **(A)**. The NMDS analysis of female donkeys’ feces across different reproductive cycles **(B)**. The horizontal axis represents the first principal component, with the percentage reflecting its contribution to the variation among samples, while the vertical axis represents the second principal component. Each point in the figure represents a sample, and samples from the same group are depicted in the same color and enclosed within a clustering circle.

Using the Bray-Curtis calculation method and weighted UniFrac distance, we performed PCoA, and the results are shown in Figure 5A. The contribution rate of the first principal component (PC1) to the detected microbiota was 19.64%, while the contribution rate of the second principal component (PC2) was 16.89% (Figure 5A). The contribution rate of the P group to the first principal component was significantly different from that of the L and NP groups (*p* < 0.01), while no significant difference was found between the L and NP groups (*p* = 0.148). Furthermore, no significant differences were observed in the contribution rates of the three groups to the second principal component (*p* > 0.05).

To further analyze the diversity of the microbiota population in each group, we validated the results using non-metric multidimensional scaling (NMDS) (Figure 5B). The stress value of the NMDS analysis was 0.079, indicating that the results were relatively reliable. Among the three groups, the community composition of the feces in the P group was significantly separated from the other two groups, with overlap observed between the NP and L groups (Table 5). This suggests that the composition of gut microbiota in pregnant female donkeys is significantly different from that in lactating and non-pregnant female donkeys. Combined NMDS and MRPP analyses revealed a highly significant difference in community structure between the L group and the other two groups (*p* < 0.05) (ANOSIM, *p* = 0.008 and 0.028; MRPP, *p* = 0.009 and 0.015; Table 6).

**Table 5:** Weighted unifrac distance in fecal microbiota of female across different reproductive cycles.

Group	L	P	NP
L	0		
P	0.242791	0	
NP	0.189491	0.284191	0

In the table, rows and columns represent samples from different groups, with the numbers indicating the dissimilarity coefficients between them. Lower values denote a smaller difference in species diversity between the two samples.

**Table 6:** Analysis results of MRPP and ANOSIM difference in fecal microbiota of female donkeys across different reproductive cycles.

Group	MRPP				ANOSIM	
	A	Observed-delta	Expected-delta	Significance	*R*-value	*p*-value
L-NP	0.03246	0.5607	0.5795	0.009	0.268	0.008
NP-P	0.03534	0.5538	0.5741	0.015	0.224	0.028
L-P	0.09905	0.4784	0.5310	0.007	0.776	0.013

Significance < 0.05 and *p*-value < 0.05 indicate a significant difference, while Significance < 0.01 and *p*-value < 0.01 represent a highly significant difference.

### 3.4 Functional predictions in the fecal microbiota of female donkeys across different reproductive cycles

**Figure 6:**
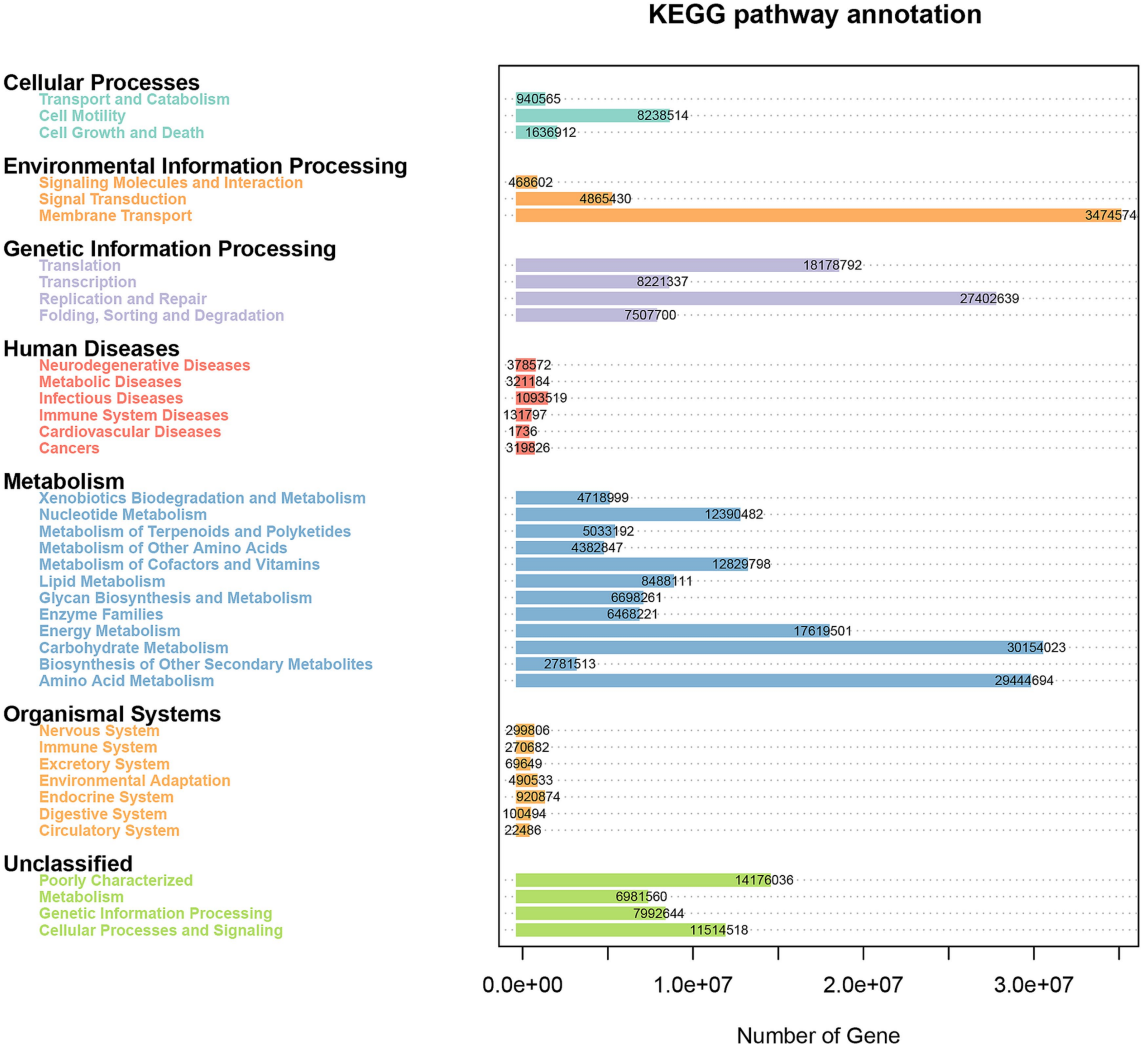
Pathway and enrichment genes number in the fecal microbiota of female donkeys across different reproductive cycles.

**Figure 7:**
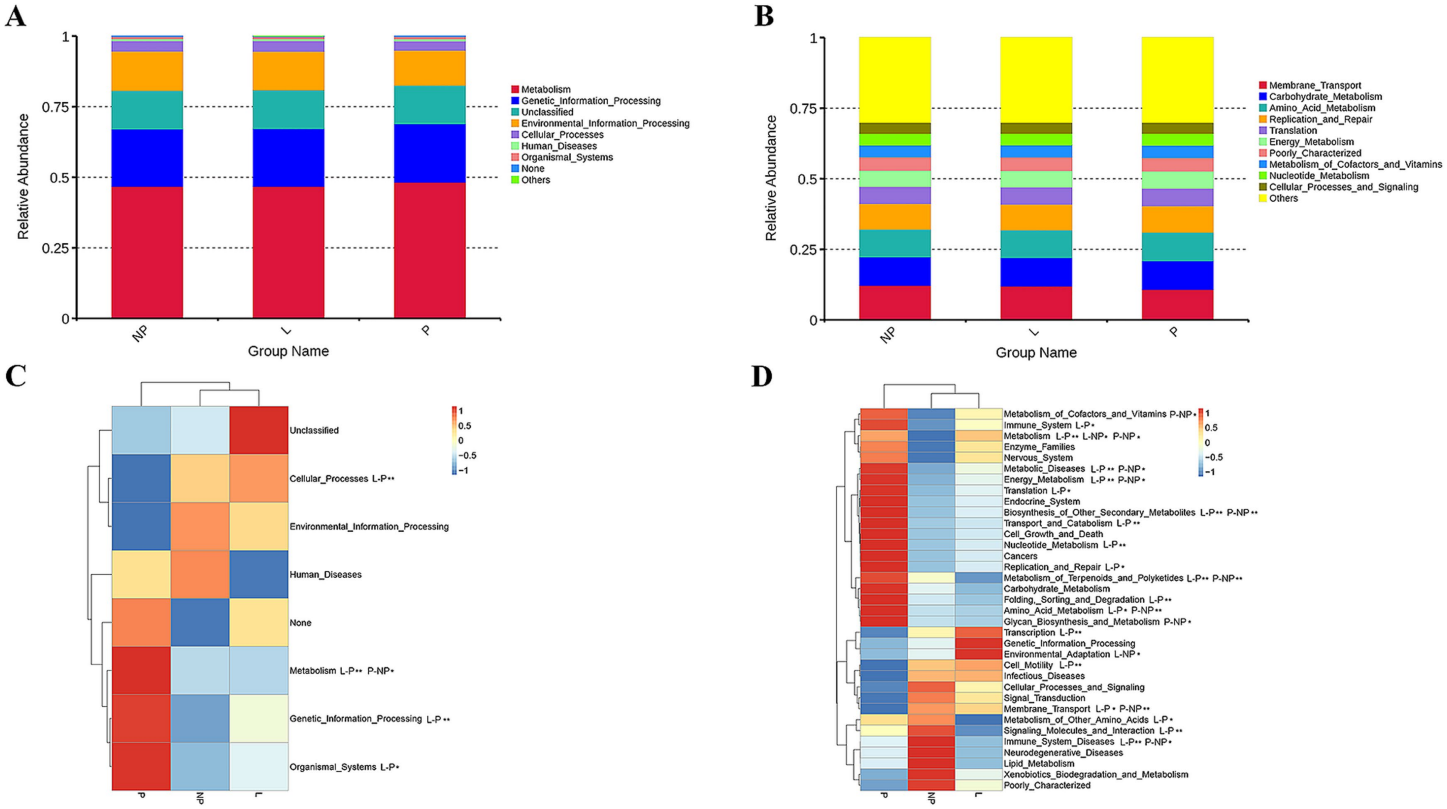
Relative enrichment of KEGG level 1 **(A)** and level 2 **(B)**. Heatmaps illustrating KEGG level 1 pathways **(C)** and KEGG level 2 pathways **(D)**. One-way ANOVA was applied to analyze differences in the relative abundance of each functional gene. *indicates significant differences (*p* < 0.05); while **indicates highly significant differences (*p* < 0.01).

We used PICRUSt to predict the functions in the fecal microbiota of female donkeys across different reproductive cycles and evaluated the accuracy of these predictions using the Nearest Sequenced Taxon Index (NSTI) values. All NSTI values derived from PICRUSt analysis were greater than 0.15. NSTI values above 0.15 indicated high prediction accuracy, whereas values below 0.06 were considered low. These values suggested a strong alignment with the reference microbial genome database, indicating high confidence in the predicted metabolic functions of the microbial communities across each lamina. Figure 6 shows that a total of 267,030,623 genes were enriched across 39 KEGG pathways. Metabolism was the most enriched level 1 pathway across all groups, while the most prominent level 2 pathways included membrane transport, carbohydrate metabolism, amino acid metabolism, and replication and repair (Figures 7A,B). Figure 7C shows that genes associated with environmental information processing were significantly lower in the P group than in the other two groups (*p* < 0.01), and genes related to cellular processes were significantly lower in the P group than in the L group (*p* < 0.01). Conversely, genes related to metabolism and genetic information processing were significantly higher in the P group than in the L group (*p* < 0.01). Additionally, metabolism-related genes and organismal system-related genes were significantly higher in the P group than in the NP and L groups, respectively (*p* < 0.05).

**Figure 8:**
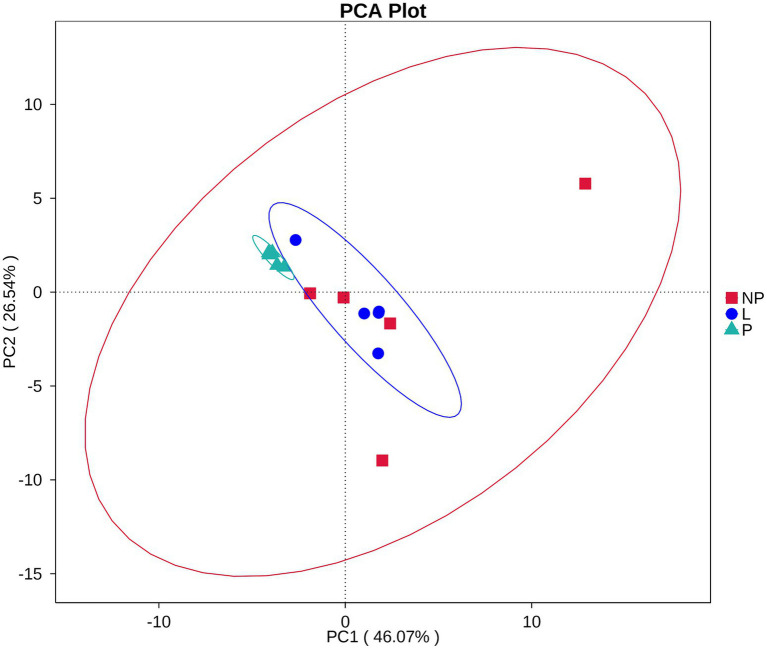
PCA of KEGG level 2 pathways in fecal microbiota of female donkeys across different reproductive cycles.

Among the 35 level 2 KEGG pathways, the relative abundance of membrane transport, carbohydrate metabolism, amino acid metabolism, translation, and energy metabolism was highest during different reproductive cycles. Figure 7D illustrates that genes involved in immune system processes, metabolism, metabolic diseases, energy metabolism, translation, biosynthesis of other secondary metabolites, transport and catabolism, cell growth and death, replication and repair, nucleotide metabolism, metabolism of terpenoids and polyketides, protein folding, storage and degradation, amino acid metabolism, and glycan biosynthesis and metabolism were significantly more expressed in the P group compared to the L or NP groups (*p* < 0.05 or *p* < 0.01). Additionally, genes related to transcription, environmental adaptation, and cell motility were expressed at significantly higher levels in the L group compared to the P group (*p* < 0.05 or *p* < 0.01). In contrast, genes associated with the metabolism of other amino acids, signaling molecules and interactions, and immune system diseases were significantly less expressed in the L group compared to the P and NP groups (*p* < 0.05 or *p* < 0.01), while genes related to membrane transport were significantly more expressed in the NP group than in the P and L groups (*p* < 0.05). PCA revealed that the functional KOs in the fecal microbiota of female donkeys across different reproductive cycles clustered, with the two components explaining a total of 72.61% of the variation. The results indicated that the functional KOs in the P group were distinctly separated from the other two groups, explaining 26.54% of the variation. The composition of the non-pregnant period showed greater individual variation, indicating more diversity in the gut microbiota of non-pregnant female donkeys (Figure 8).

## 4 Discussion

This study examines the gut microbial ecosystems within the fecal microbiota of female donkeys across different reproductive cycles. Upon examining the bacterial OTUs shared across these three periods, we identified 2,117 common OTUs, with each group also harboring its own unique OTUs. The presence of OTUs common to all groups suggests a similar bacterial community structure, while also reflecting the specificity of the microbial DNA components. Additionally, we observed that the microbial diversity in the feces of pregnant donkeys was notably more variable than in other breeding cycles. The fecal microbiota of female donkeys is rich and diverse, containing a large number of bacterial phyla, which is consistent with studies on the fecal microbiota structure and composition of horses (Lv et al., 2023).

However, there were no significant differences in the gut microbial alpha diversity and richness indices across different reproductive cycles in female donkeys, indicating that the reproductive cycle has no significant impact on the quantity and diversity of the microbiota. This finding contrasts with the observed continuous increase in alpha diversity in sows from pregnancy to the lactation and weaning stages (Liu H. et al., 2019). Many factors can influence the alpha diversity of gut microbiota, such as feed, climate, and geographical location. Ji and co-authors observed that the alpha diversity of sow gut microbiota increased from pregnancy to weaning, with the different stages of pregnancy significantly affecting the community structure (Ji et al., 2019). Temperature fluctuations may alter the optimal growth range of gut microbiota, thereby affecting the composition and functional expression of the microbial community. Seasonal variations can also influence immune function through hormones such as melatonin and cortisol, regulating the host’s selective pressure and barrier function toward microorganisms. Given the close association between the gut microbiota and the host immune system, seasonal immune changes may contribute to microbial fluctuations. In addition, seasonal shifts in forage type and nutritional composition alter the available substrates in the gut, which in turn reshape the structure of the microbial community. The difference in results may be due to variations in timing and climate; this experiment was conducted at the end of June, while the aforementioned study was carried out in late October. Dietary changes significantly influence the gut microbiota (Perler et al., 2023), implying that seasonal dietary variations may introduce confounding effects in this study. Other studies have found no significant differences in alpha richness indices (ACE and Chao) or diversity indices (Shannon and Simpson) of the gut microbiota in sows at 30 and 90 days of pregnancy (Xue et al., 2017).

A key function of symbiotic gut microbiota is to aid in nutrient absorption. While animals can enzymatically digest proteins, lipids, and simple sugars, their enzymes are incapable of breaking down the complex structural polysaccharides in plants (Yoshimura et al., 2024). Microbes fill this gap by converting these polysaccharides into volatile fatty acids, which the host can absorb and utilize as an energy source (Houtman et al., 2022). The gut microbiota of female donkeys across different reproductive cycles is predominantly composed of *Firmicutes* and *Bacteroidetes*, consistent with previous findings on the intestinal microbial composition in donkeys (Liu G. et al., 2019; Liu H. et al., 2019; Xing et al., 2020). The average ratio between *Firmicutes* and *Bacteroidetes* in the fecal microbiota of pregnant, non-pregnant, and lactating female donkeys is 0.9:1, 1.8:1, and 1.7:1, respectively. *Firmicutes* primarily degrade cellulose, while *Bacteroidetes* are responsible for breaking down carbohydrates, reflecting the trade-offs between carbohydrate and protein fermentation. This study revealed that the abundance of *Firmicutes* in the gut of lactating female donkeys was significantly higher than during pregnancy or the non-pregnant periods (*p* < 0.05). In contrast, the abundance of *Bacteroidetes* was significantly higher during pregnancy compared to the lactation and non-pregnant periods (*p* < 0.05). This is consistent with reports that the relative abundance of *Anaerofustis*, *Bacteroidetes*, and *Ruminococcaceae* in the feces of Tibetan antelopes significantly increases during late pregnancy compared to the postpartum period (Shi et al., 2021). It is known that the ratio of *Firmicutes* to *Bacteroidetes* significantly affects fat deposition in the body (Magne et al., 2020). During pregnancy and lactation, animals’ metabolic demands significantly increase, and the composition of their microbiota also changes. *Firmicutes* play an important role in energy extraction, helping the host more efficiently obtain energy from food. In contrast, an increase in *Bacteroidetes* may promote fat metabolism and gut health, improve nutrient absorption, and reduce excessive energy storage. Gasmi Benahmed et al. (2021) noted that the host’s ability to store energy after food intake increases with a higher *Firmicutes/Bacteroidetes* ratio in the gut. Lactating female donkeys adapt to lactation and body condition recovery through changes in their gut microbiota (Gasmi Benahmed et al., 2021). Pregnant female donkeys typically experience a physiological decline in insulin sensitivity, especially during mid to late gestation, marked by a mild increase in insulin resistance to ensure sufficient glucose supply for the developing fetus. To some extent, an increase in *Bacteroidetes* can enhance the efficiency of nutrient utilization in the gut, promote fat mobilization and energy redistribution, rather than excessive energy storage. The rise in *Bacteroidetes* may reflect a microbial adaptation to these changes, facilitating lipid metabolism and improving nutrient absorption to help the mother maintain metabolic balance under insulin-resistant conditions. We also found that the abundance of *Alloprevotella* in the feces of pregnant donkeys was significantly higher than that in lactating and non-pregnant donkeys (*p* < 0.01). *Alloprevotella* can provide energy by degrading dietary fiber (Wang et al., 2022). A higher proportion of *Firmicutes* is associated with increased energy storage in the body, whereas a greater proportion of *Bacteroidetes* is generally linked to improved metabolic health. A higher proportion of *Firmicutes* is associated with increased energy storage in the body, whereas a greater proportion of *Bacteroidetes* is typically linked to a healthier gut environment and better metabolic health. Therefore, it is speculated that the gut microbiota of pregnant and lactating female donkeys supplies more energy to the body compared to donkeys in other reproductive stages, supporting pregnancy maintenance and nursing foals. The gut microbiota of pregnant female donkeys plays a key role in energy metabolism. During the feeding process, optimizing management practices can help enhance the diversity and health of the gut microbial community. At the same time, in breeding programs, it is advisable to select female donkeys with a more diverse gut microbiota and a greater ability to regulate energy metabolism effectively. The interaction between host genotype and gut microbiota may influence metabolic health during pregnancy, and selecting individuals with strong adaptability and a healthy gut profile may improve reproductive performance during gestation.

Research has found that the maternal gut microbiota undergoes significant changes during pregnancy (Fu et al., 2024). However, recent studies have indicated contradictory findings, suggesting that the gut microbiota remains remarkably stable throughout pregnancy (Avershina et al., 2014). The *β*-diversity analysis of gut microbiota revealed that the composition and structure of gut microbiota in lactating donkeys were more similar to those in non-pregnant donkeys (*p* < 0.05). This result differs from findings in sows, where the gut microbiota during pregnancy was more similar to that during the non-pregnant period than during lactation (Liu H. et al., 2019). This discrepancy could be due to the uniform feeding regimen in our study, as opposed to the dietary changes implemented during lactation in another research. Compared to other species, research on the effects of different reproductive cycles on the composition and structure of gut microbiota in donkeys is relatively scarce. Analysis of fecal microbial differences in female donkeys across reproductive cycles revealed that unidentified_*Bacteroidales* and *Alloprevotella* in pregnant donkeys, along with unidentified_*Clostridiales*, *Terrisporobacter*, and *Sarcina* in lactating donkeys, may be linked to pregnancy or lactation. Previous studies have shown that *Bacteroides* is positively correlated with human weight gain and glucose intolerance, suggesting its potential role in energy storage. This is consistent with our earlier observation that the gut microbiota of pregnant donkeys provides additional energy to support pregnancy maintenance and fetal development. A normal and healthy pregnancy is characterized by a systemic inflammatory response in the mother, with a sharp increase in white blood cells and intracellular reactive oxygen species, and the body’s anti-inflammatory response remains high until lactation (Horvat Mercnik et al., 2024). Research has shown that members of *Clostridium* species in the gut microbiota of mouse models of colorectal cancer (CRC) are associated with lower tumor burdens (Montalban-Arques et al., 2021). The research also showed that the feces of lactating female donkeys contain higher abundances of *Terrisporobacter* and *Sarcina*, which have pathogenic potential. This bacterial genus has been linked to gastrointestinal inflammation and metabolic dysregulation. It may trigger immune responses through its metabolites and contribute to lipid metabolic abnormalities or oxidative stress. Additionally, these microbial alterations could influence milk composition via metabolite-mediated pathways, potentially impacting the health of the foals. Therefore, we speculate that postpartum conditions may promote the colonization of pathogenic microorganisms while reducing the presence of beneficial symbiotic microbes. Overall, the changes in the gut microbial composition of female donkeys from pregnancy to lactation periods reflect the metabolic and immune adaptations during these critical transitional phases. We recommend regular monitoring of the gut microbiota composition in lactating donkeys, with particular attention to changes in potential pathogenic bacteria, to facilitate early detection of microbial dysbiosis. Additionally, supplementing the maternal diet with probiotics may help regulate gut microecology, enhancing microbial diversity and stability.

The KEGG pathway database is one of the most widely used bioinformatics resources, commonly used in functional annotation and metabolic pathway analysis. KEGG provides a systematic mapping from genes to metabolic pathways, allowing predicted functional genes to be categorized into specific biological processes or pathways. Therefore, it is the preferred database for functional prediction based on 16S rRNA data. PICRUSt functional prediction analysis indicated that metabolism (carbohydrate metabolism and biosynthesis of other secondary metabolites) and genetic information processing (such as replication and repair, translation) were prominent in the fecal microbiota of female donkeys across different reproductive cycles. We found that transcription, environmental adaptation, and cell motility were more enriched in lactating female donkeys, while metabolism of other amino acids and immune system diseases were significantly less expressed. The immune system and metabolism-related genes of pregnant female donkeys were significantly expressed, confirming the strength of the maternal immune system and the high metabolic activity during pregnancy. The functional variations in the gut microbiota of female donkeys across different reproductive cycles suggest a weakened immune system in lactating donkeys during the postpartum period. These microbial adaptations may enhance environmental responsiveness, underscoring the need for sufficient nutritional support to meet the demands of postpartum donkeys. Additionally, the dynamic interplay between the host and microbiota drives continuous changes in microbial functions throughout the host’s lifespan (Culp and Goodman, 2023), which aligns with our findings. The 35 level 2 KEGG pathways among female donkeys of different reproductive cycles showed significant differences, suggesting that reproductive cycles are linked to variations in the composition, diversity, and function of fecal microbiota, as the microbiota responds to rapid changes in the body to adapt to new environments. Pregnant female donkeys demonstrate significantly elevated expression levels of immune system activity, metabolic pathways, and associated genes compared to lactating female donkeys. This highlights the robust maternal immune system and the intensified metabolic demands characteristic of the pregnancy period. These findings suggest that both the composition and functionality of the gut microbiota adapt as female donkeys progress through different reproductive cycles, with environmental factors and available resources influencing the shaping of the microbial community. The significantly altered pathways identified across different reproductive cycles in female donkeys may represent potential targets for future research, suggesting possible changes in microbial functional activity under varying conditions. However, these findings require further validation through functional gene expression and related analyses.

## 5 Conclusion

In summary, we analyzed the fecal microbiota of female donkeys across different reproductive cycles using high-throughput 16S rRNA gene sequencing and PICRUSt for functional prediction. Our findings indicate that the alpha diversity index of fecal microbiota in female donkeys showed no significant differences across different reproductive cycles. However, the microbial composition varied significantly: *Firmicutes* were notably higher during lactation, while *Bacteroidetes* were significantly more abundant during pregnancy. *Bacteroidetes* and *Alloprevotella* were dominant during pregnancy in donkeys, while *Firmicutes* and unidentified *Clostridiales* prevailed during lactation. By correlating metabolic functions with microbial phyla, we infer that the metabolic and immune functions of the gut microbiota in lactating donkeys are weaker compared to those during pregnancy. These findings deepen our understanding of the variations in fecal microbiota across reproductive cycles in female donkeys. However, further studies are needed to explore in greater detail the relationships between microbial changes and metabolism during reproductive cycles. PICRUSt functional prediction analysis revealed a potential decline in immune function in lactating female donkeys. Accordingly, targeted supplementation with probiotics during production management may help strengthen their immune responses.

## Data Availability

The datasets presented in this study can be found in online repositories. The names of the repository/repositories and accession number(s) can be found at: https://www.ncbi.nlm.nih.gov/, PRJNA1170075.
