# Correction: The composition and predictive function of the fecal microbiota in female donkeys across different reproductive cycles

**DOI:** 10.3389/fmicb.2025.1658709

**Published:** 2025-08-08

**Authors:** Jingya Xing, Mingquan Jia, Guoliang Zhang, Lanjie Li, Shuai Liu, Guangyu Li, Guiqin Liu

**Affiliations:** ^1^College of Animal Science and Technology, Qingdao Agricultural University, Qingdao, China; ^2^College of Agronomy, Liaocheng University, Liaocheng, China; ^3^Laboratory of Gastrointestinal Microbiology, National Center for International Research on Animal Gut Nutrition, Nanjing Agricultural University, Nanjing, China

**Keywords:** female donkey, different reproductive cycles, fecal microbiota, microbial communities, functional prediction

In the published article, there was an error with the wording of the Funding statement:

“This work received support from the National Key Research and Development Program Project (2024YFD1300500) and the Natural Science Foundation of Shandong (ZR2024QC125).”

The correct Funding statement appears below.

“The author(s) declare that financial support was received for the research and/or publication of this article. This work received support from National Key R&D Program of China (2024YFD1300500) and Natural Science Foundation of Shandong (ZR2024QC125).”

The original article has been updated.

